# Induced Pluripotent Stem Cells for Ischemic Stroke Treatment

**DOI:** 10.3389/fnins.2021.628663

**Published:** 2021-05-28

**Authors:** Ranran Duan, Yang Gao, Ruya He, Lijun Jing, Yanfei Li, Zhe Gong, Yaobing Yao, Tingting Luan, Chaopeng Zhang, Li Li, Yanjie Jia

**Affiliations:** ^1^Department of Neurology, The First Affiliated Hospital of Zhengzhou University, Zhengzhou, China; ^2^The International Medical Center, The First Affiliated Hospital of Zhengzhou University, Zhengzhou, China; ^3^Department of Anesthesiology, Beijing Friendship Hospital, Capital Medical University, Beijing, China

**Keywords:** stem cells, ischemic stroke, cell therapy, treatment, mechanism

## Abstract

Ischemic stroke is one of the main central nervous system diseases and is associated with high disability and mortality rates. Recombinant tissue plasminogen activator (rt-PA) and mechanical thrombectomy are the optimal therapies available currently to restore blood flow in patients with stroke; however, their limitations are well recognized. Therefore, new treatments are urgently required to overcome these shortcomings. Recently, stem cell transplantation technology, involving the transplantation of induced pluripotent stem cells (iPSCs), has drawn the interest of neuroscientists and is considered to be a promising alternative for ischemic stroke treatment. iPSCs are a class of cells produced by introducing specific transcription factors into somatic cells, and are similar to embryonic stem cells in biological function. Here, we have reviewed the current applications of stem cells with a focus on iPSC therapy in ischemic stroke, including the neuroprotective mechanisms, development constraints, major challenges to overcome, and clinical prospects. Based on the current state of research, we believe that stem cells, especially iPSCs, will pave the way for future stroke treatment.

## Introduction

Stroke is one of the major central nervous system diseases associated with high disability and mortality rates, approximately 80% of the cases being ischemic stroke (Phipps and Cronin, 2020). Although there are many clinical treatment methods available currently for patients with ischemic stroke, including antiplatelet aggregation, anticoagulation, improving microcirculation, and improving brain metabolism, these do not have efficient regeneration and repair effects on the necrotic nerve cells in the ischemic area. The standard treatment method is to either use recombinant tissue plasminogen activator (rt-PA) or perform mechanical thrombectomy to restore blood flow. However, these treatments have certain limitations. The effective treatment time window of rt-PA is 4.5 h, and patients with large artery occlusion can be treated with thrombectomy within 6 h or even beyond 24 h of symptom onset. However, nearly 60% of patients did not achieve functional independence at 3 months after treatment in recent mechanical thrombectomy trials (Rabinstein, 2020; Shafie and Yu, 2021). When ischemic stroke occurs, apoptosis or necrosis of various cells is seen in the infarct areas. Therefore, the ideal treatment method would be to implant a certain type of cell that can replace the damaged cells. In recent years, stem cell transplantation technology has been successfully applied in heart disease modeling (Musunuru et al., 2018), diabetes mellitus (Kalra et al., 2018), macular degeneration (Bracha et al., 2017), spinal cord injury (Csobonyeiova et al., 2019), cartilage regeneration (Castro-Viñuelas et al., 2018), and graft-vs.-host disease (Bloor et al., 2020). Stem cell transplantation is also being gradually considered for the treatment of some nervous system diseases, such as Alzheimer’s disease (Robbins and Price, 2017), Parkinson’s disease (Cobb et al., 2018), amyotrophic lateral sclerosis (Csobonyeiova et al., 2017), Huntington’s disease (Tousley and Kegel-Gleason, 2016; Yoon et al., 2020), and ischemic stroke. In this review, we have summarized the progress that has been made in the development of cell therapy for ischemic stroke. We have particularly focused on the mechanism, current status, clinical application, development constraints, and future prospects of the application of induced pluripotent stem cells (iPSCs) in treating ischemic stroke.

## Current Applications of Stem Cells

Stem cell transplantation technology is attractive as a novel option for the treatment of ischemic stroke, and utilizes different cell sources, including embryonic stem cells (ESCs), neural stem cells (NSCs), mesenchymal stem cells (MSCs), umbilical cord-derived blood cells, adipose-derived MSCs, dental stem cells, iPSCs, and some immortalized cell lines; the most recent studies in this field have primarily focused on the first three stem cell types. Here, we have discussed the current applications of different stem cell types in ischemic stroke, especially the iPSC therapy.

## Embryonic Stem Cells (ESCs) in Ischemic Stroke

ESCs, a type of pluripotent cells, are derived from the inner mass of preimplantation embryo and have differentiation ability (Thomson et al., 1998). They are able to form specific nerve tissues, such as neurons, astrocytes, and oligodendrocytes (Wichterle et al., 2002). Therefore, ESCs have been regarded as a potential and ideal source of transplanted cells for neural disease therapy. Cell therapy based on ESCs has been shown to not only promote structural repair and functional recovery but also reduce the infarct size in a mouse model of ischemic stroke. After transplanting mouse ESCs into a severe focal ischemic rat cortex, ESC-derived cells expressing cell surface markers of neurons, astrocytes, oligodendrocytes, and endothelial cells could be found in the lesion area; moreover, the survival rate, neuronal differentiation, structural repair, and functional outcome were further improved by transplantation of ESCs overexpressing Bcl-2. Additionally, the intracerebral transplantation of mouse ESCs could have a positive effect on motor and sensory recovery and infarct size reduction in rats with focal ischemia (Wei et al., 2005; Nagai et al., 2010; Tae-Hoon and Yoon-Seok, 2012). Some studies have found nerve cells derived from human ESCs to be at risk of malignant transformation and teratoma formation, which is possibly caused by the stimulation of various local cytokines (Brederlau et al., 2006; Seminatore et al., 2010). From an ethical point of view, the limited sources and the associated high incidence of malignant transformation restrict the wide application of ESCs. Therefore, research on ESC application in stroke treatment is very limited.

## Neural Stem Cells (NSCs) in Ischemic Stroke

Neurogenesis was not possible until the discovery of NSCs, which have the ability to renew themselves and originate neurons, astrocytes, and oligodendrocytes (Reynolds and Weiss, 1992). Many studies have identified the existence of multipotent and self-renewing NSCs in different areas of the brain, including the subgranular zone in the dentate gyrus of the hippocampus and subventricular zone of the lateral ventricles, and the occurrence of endogenous neurogenesis in the adult brain (Gage, 2000; Koh and Park, 2017). Researchers have found that grafting of fetal neocortical tissue 1 week after focal brain ischemia improved the behavioral outcome and reduced secondary thalamic atrophy (Mattsson et al., 1997). Further research showed that transplanted human fetal NSCs were able to survive, migrate, and differentiate in ischemic stroke-damaged rats (Kelly et al., 2004; Darsalia et al., 2007). Human fetal NSCs were implanted into the ipsilateral striatum of a middle cerebral artery occlusion (MCAO) rat model 48 h after model establishment, and behavioral assessments were conducted at 6 and 14 weeks. Results showed that the grafted human NSCs survived in all rats and successfully differentiated to neuroblasts or mature neurons; functional recovery of the rats grafted with NSCs also improved at 6 and 14 weeks, including sensorimotor and spatial learning functions (Mine et al., 2013). Moreover, long-term survival and widespread distribution of NSCs were detected following intra-arterial delivery in an ischemic stroke rodent model, and cell differentiation was evident at 4 weeks (Zhang et al., 2020). In a cortical stroke model, stable graft survival and neuronal differentiation were successfully monitored, and human NSC transplantation was found to have a profound effect on network stability (Minassian et al., 2020). In addition, neural progenitor cells derived from human iPSCs were found to be more effective than mesenchymal stromal cells obtained from human placenta in suppressing the progression of experimental ischemic stroke by improving animal survival in the most acute period and accelerating the recovery of neurological deficit and body weight (Cherkashova et al., 2019). Rats that received pretreatment with human iPSC-NSCs and metformin recovered and had a faster decrease in infarct volume compared to the controls (Ould-Brahim et al., 2018). Furthermore, studies have shown that neuron stem cell extracellular vesicles (NSC EVs) could improve tissue and functional recovery in both rat and pig models of ischemic stroke, and possessed therapeutic potential (Webb et al.,2018a,b; Spellicy et al., 2020). However, NSC death or cell fate switch may happen in the case of hypoxia and ischemia, caused by a change in cell metabolism and irreversible switch from neurogenesis to gliosis via enhanced Notch signaling (Santopolo et al., 2020). Additionally, other obstacles also hinder NSC use in ischemic stroke treatment, including the limited resources with ethical significance, insufficient biological understanding, minimal clinical exploration, and risk of tumorigenesis when derived from iPSCs (Alessandrini et al., 2019).

## Mesenchymal Stem Cells (MSCs) in Ischemic Stroke

MSCs are non-hematopoietic stem cells, which widely exist in various organs and tissues, and have a high degree of proliferation, along with self-renewal and multi-directional differentiation properties. MSCs can be obtained from several tissues, including the bone marrow, placenta, amniotic fluid, adipose tissue, umbilical cord, and dental pulp (Yalvac et al., 2009; Yang et al., 2011; Pittenger et al., 2019). At the preclinical level, transplantation of MSCs, especially BMSCs, improved functional recovery in MCAO model rats. MSC transplantation was shown to reduce the lesion volume (Koh et al., 2008; Li et al., 2010; Shen et al., 2011), enhance sensorimotor (Huang et al., 2013) and cognitive functions (Lowrance et al., 2015), promote angiogenesis and neurogenesis (Bao et al., 2011), modulate inflammatory and immune responses (Ohtaki et al., 2008), and improve synaptic recovery (Asgari Taei et al., 2021). Although some studies have shown no significant reduction of ischemic lesion volumes, the functional outcome nevertheless improved after the administration of MSCs; these discrepancies were suggested to be due to not recanalizing the middle cerebral artery (Zacharek et al., 2010; Gutiérrez-Fernández et al., 2013). Several clinical trials seem to have confirmed MSCs to be a potential option for the treatment of ischemic stroke; moreover, MSC therapy was demonstrated to be safe for subacute stroke patients in the long-term and may improve recovery after stroke according to a randomized controlled trial using an intravenously applied MSC culture expanded with fetal bovine serum (Lee et al., 2010; Díez-Tejedor et al., 2014; Steinberg et al., 2016; Levy et al., 2019; Lalu et al., 2020). Furthermore, MSC-derived extracellular vesicles (MSC EVs) played an important role in ischemic stroke as a treatment and potential biomarker (Otero-Ortega et al., 2019). However, no efficacy of MSC therapy was observed in the only randomized Phase II study until now (Hess et al., 2017). Hence, the efficacy of MSCs for stroke treatment remains to be determined.

## Induced Pluripotent Stem Cells (iPSCs) in Ischemic Stroke

iPSCs form a class of cloned cells with characteristics similar to those of ESCs, and were first discovered by Takahashi and Yamanaka using retroviruses to integrate four factors (Sox2, Oct3/4, Klf4, and cMyc) into both mouse and adult human fibroblasts through corresponding vectors (Takahashi and Yamanaka, 2006; Takahashi et al., 2007). Till date, iPSCs have mainly been applied in three aspects, namely disease modeling, drug discovery, and regenerative therapy (Wilson and Wu, 2015). In recent years, iPSC-based cell therapy has developed rapidly and its potential has been studied for the treatment of many diseases. Retinal pigment epithelial cells differentiated from iPSCs have proven to be safe and effective in both preclinical models and clinical studies of macular degeneration (Li et al., 2012; Kamao et al., 2014; Yoshida et al., 2014; Sharma et al., 2019). As a novel alternative method, iPSC technology has attracted increasing attention in various other diseases, including ischemic stroke, heart failure, hematopoietic disorders, spinal cord injury, and liver disease (Lachmann et al., 2015; Hansel et al., 2016; Miyagawa and Sawa, 2018; Fernández-Susavila et al., 2019; Trawczynski et al., 2019). Since a detailed description of all these applications is beyond the scope of this review, we have focused specifically on the key issues related to ischemic stroke.

A variety of animal experiments have shown that the application of iPSCs plays an important role in ischemic stroke treatment, including improving sensorimotor function (Chen et al., 2010; Jiang et al., 2011; Gomi et al., 2012; Oki et al., 2012; Tatarishvili et al., 2014; Eckert et al., 2015; Lau et al., 2018; Oh et al., 2020), reducing lesion volume (Chen et al., 2010; Baker et al., 2017; Oh et al., 2020; Xia et al., 2020), promoting neurogenesis and angiogenesis (Oki et al., 2012; Oh et al., 2020), and exerting immunomodulatory and anti-inflammatory effects in the brains of rodents affected by ischemic stroke (Eckert et al., 2015). However, some animal experiments did not achieve the desired results, such as a significant reduction in infarct volume and improvement in behavior (Jensen et al., 2013; Kawai et al., 2010). Here, a summary of iPSC transplantation experiments in ischemic stroke models is shown in Table 1, and summarized the animal models of ischemic stroke, follow-up period and experimental results.

**TABLE 1:** Summary of iPSC transplantation experiments in ischemic stroke models.

Model (reference)	Follow-up period	Results
MCAO rat (Xia et al., 2020)	2–28 days	iMSC-sEVs migrate to the brain, infarct size ↓, mNSS↓, error step number in the foot-fault test ↓, overall blood vessel density↑
MCAO rats (Jiang et al., 2011)	4–16 days	iPSCs migrate to the ischemic brain and differentiate into neural cells, no seizure or convulsive activity,%HLV↓, sensorimotor function↑
MCAO rats (Chen et al., 2010)	1–4 weeks	iPSCs-FG differentiate into astroglial-like and neuron-like cells, infarct size↓, motor function↑, anti-inflammatory cytokines ↑, pro-inflammatory cytokines ↓ but form teratoma in 4 weeks
Pig (Baker et al., 2017)	24 h to12 weeks	CBV, white matter integrity, neurometabolite abundance (NAA, Cr, Cho) ↑
Mouse (Gomi et al., 2012)	1–6 weeks	iPSCs differentiate into neuronal progenitors, axonal elongation, mNSS ↓ but immune rejection still exists after 6 weeks
MCAO pig (Lau et al., 2018)	1–12 weeks	Recovery of postural reactions, posture, mental status, and appetite
MCAO rats (Oki et al., 2012)	1–10 weeks	Extend axons to the GP, VEGF and recovery of fine forelimb movements ↑
MCAO rats (Tatarishvili et al., 2014)	1–8 weeks	Behavioral recovery ↑
MCAO rats (Eckert et al., 2015)	1–30 days	Behavioral recovery ↑, BBB leakage ↓, pro-inflammatory cytokine ↓, microglial activation ↓, adhesion molecules ↓, MCP-1 and MIP-1α↓
MCAO rats (Oh et al., 2020)	1–12 weeks	Ep-iPSC-NPCs differentiated into neuronal and glial cells, sensorimotor and behavioral functional recovery ↑, MEP ↑, proliferating and migrating neural precursors ↑, astroglial scar formation ↓, microglial ↓, mNSS ↓, final infarct size ↓
MCAO rats (Jensen et al., 2013)	1–5 weeks	NSCs derived from human iPSCs survive and differentiate into neural cells, but without behavioral recovery or reduction of infarct size
MCAO rat (Kawai et al., 2010)	1–28 days	No significant difference in behavioral recovery and form larger tumors than the sham-operated group
MCAO rat (Tornero et al., 2017)	48 h to 6 months	iPSCs promoted synapse formation between neurons, and the grafted neurons received direct synaptic inputs from neurons

*BBB, blood-brain barrier; CBV, cerebral blood volume; Cr, creatine; Cho, choline; GP, globus pallidus; iPSC, induced pluripotent stem cell; MSC, mesenchymal stem cell; MCAO, middle cerebral artery occlusion; iMSC-sEV, small extracellular vesicles secreted by MSCs derived from human iPSCs; mNSS, modified neurological severity score;%HLV, percentage hemisphere lesion volume; iPSC-FG, iPSCs with fibrin glue; MEP, motor-evoked potential; MCP-1, monocyte chemotactic protein 1; MIP-1α, macrophage inflammatory protein 1α; NAA, N-acetylaspartate; ep-iPSC-NPCs, neural precursor cells derived from human-iPSCs through episomal plasmid-based reprogramming; VEGF, vascular endothelial growth factor.*

### Mechanism of Action of iPSCs in Ischemic Stroke

**FIGURE 1:**
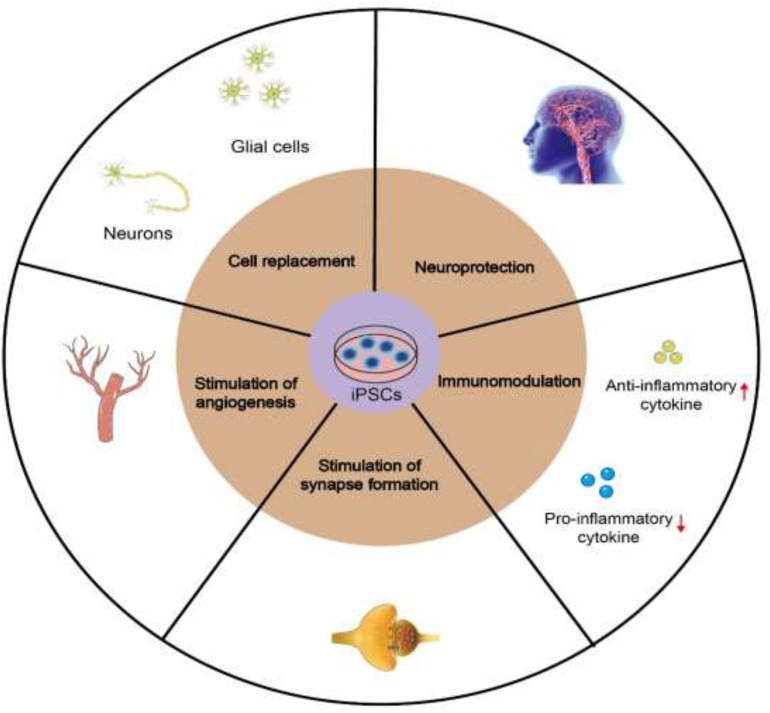
Mechanisms of iPSCs in treating ischemic stroke, including cell replacement, neuroprotection, stimulation of angiogenesis, synaptogenesis and endogenous neurogenesis, and modulation of inflammatory and immune responses.

Neurological deficit caused by ischemic stroke mainly occurs due to the loss of various nerve cells, including neurons and different types of glial cells. Implantation of iPSCs in animal models of ischemic stroke can effectively promote the recovery of nerve function. Multiple mechanisms have been proposed to account for these beneficial effects of iPSCs in treating ischemic stroke, including cell replacement, neuroprotection, modulation of inflammatory and immune responses, and stimulation of angiogenesis, synaptogenesis and endogenous neurogenesis (Figure 1).

#### Cell Replacement and Neuroprotection

iPSC-derived NSCs (iNSCs), including iPSCs generated by an episomal plasmid-based reprogramming technique, can differentiate into the three major neural lineages, namely neurons, astrocytes, and oligodendrocytes (Baker et al., 2017; Oh et al., 2020). The microenvironment may be a potential factor contributing to the differentiation potential of iPSCs (Jiang et al., 2011). Baker et al. (2017) had found no significant difference in the number of neurons at the margin of the lesion between the iNSC-treated group and normal control animals 12 weeks after transplantation, whereas the number of neurons in the non-treated group was lower than that in normal control animals. Moreover, the expression levels of genes related to angiogenesis, neurotrophism, and inflammation were significantly altered in the brain tissues of the iNSC-treated group. Non-invasive longitudinal magnetic resonance imaging of stroke model animals provided evidence of brain metabolism recovery, white matter integrity, and cerebral blood perfusion after iNSC therapy at the tissue level. Another study found the transplantation of human iPSC-derived long-term expandable neuroepithelial-like stem cells (It-NESCs) into the striatum of stroke-injured rats to improve behavioral recovery, which could be observed in the early stages after transplantation (Oki et al., 2012). Importantly, hiPSC-lt-NESCs were shown to survive and differentiate to neurons in aged rats with ischemic stroke and further improve functional recovery (Tatarishvili et al., 2014). Together, these data suggested that iNSC treatment may have a neuroprotective effect, leading to reduced neuronal cell death in the cerebral cortex and inhibition of microglial activation, resulting in changes in gene expression that diminish inflammation and improve tissue recovery.

#### Modulation of Inflammatory and Immune Responses

Chen et al. (2010) had detected changes in inflammatory cytokine levels in the brain homogenate of MCAO model rats, 1 week after iPSC transplantation, using enzyme-linked immunosorbent assay. They found the levels of anti-inflammatory cytokines interleukin (IL)-4 and IL-10 in the brains of rats treated with subdural iPSCs cultured with fibrin glue (iPSC-FG) to be significantly higher than those of the simple cerebral ischemia control group, 1 week after subdural transplantation, whereas the levels of pro-inflammatory cytokines (IL-1β, TNF-α, IL-2, and IL-6) were significantly reduced. These results suggested that subdural-transplanted iPSCs-FG could have a possible paracrine effect, and may promote neuroprotection by increasing anti-inflammatory cytokines and decreasing pro-inflammatory cytokines. Eckert et al. (2015) had also supported the effect of early transplantation on host immune response, showing that transplantation of iPSC-derived NSCs into the hippocampus 24 h after stroke could attenuate the blood-brain barrier damage while reducing the expression levels of pro-inflammatory markers, microglial activation, and adhesion molecules, resulting in significant improvement of motor and sensorimotor functions in the first week after transplantation.

#### Stimulation of Angiogenesis

Vascular endothelial growth factor (VEGF) expression was detected in astrocytes and in the blood vessel wall of the damaged brain. The immune reactivity of VEGF was upregulated as early as 1 week after transplantation. Although the reactivity was relatively weak at 8 weeks after transplantation in aged rats, the result suggested that VEGF secretion has a certain effect on plasticity and inflammation in early recovery (Oki et al., 2012; Tatarishvili et al., 2014). Direct implantation of cells expressing the VEGF receptor fetal liver kinase-1, derived from iPSCs, into the ischemic area could effectively promote the formation of new blood vessels (Suzuki et al., 2010). In addition, the small extracellular vesicles secreted by MSCs derived from human iPSCs had the ability to promote angiogenesis and provide protection against brain injury after ischemic stroke by inhibiting signal transducer and activator of transcription 3 (STAT3)-dependent autophagy (Xia et al., 2020).

#### Stimulation of Synaptogenesis and Endogenous Neurogenesis

Further, the possibility of neural circuit reconstitution in the ischemic brain was investigated. Results indicated that iPSCs can differentiate into human telencephalic progenitors under serum-free culture of embryoid body-like aggregates, and these cells not only survived *in vivo* but also grew axons and extended to the cerebral ischemic area of the mice. iPSCs promoted synapse formation between host neurons, as demonstrated by fluorogold and synaptophysin staining of the host brain, and regulated the activity of transplanted neurons (Gomi et al., 2012; Tornero et al., 2017). In studies of human-to-human transplantation, neurons derived from PSCs were confirmed to have integrated into the adult host neural network (Grønning Hansen et al., 2020). Positive co-staining of presynaptic vesicle markers in some transplanted cells indicated these cells to participate in synaptic transmission. The transplanted cells also enhanced endogenous brain repair, including subventricular zone neurogenesis, reduction of inflammation, and formation of glial scars (Oh et al., 2020).

However, there was a lack of functional improvement and infarct area reduction, which were attributed to multiple potential factors, including the optional transplant timing after ischemic stroke, optional cell dose, and many variables of the cells themselves, such as the source, culture protocol, and differentiation stage, or other unknown reasons, suggesting that the transplanted cells need more time to mature and integrate into the neural network (Wechsler et al., 2009; Jensen et al., 2013).

### Development Constraints and Possible Solutions

**FIGURE 2:**
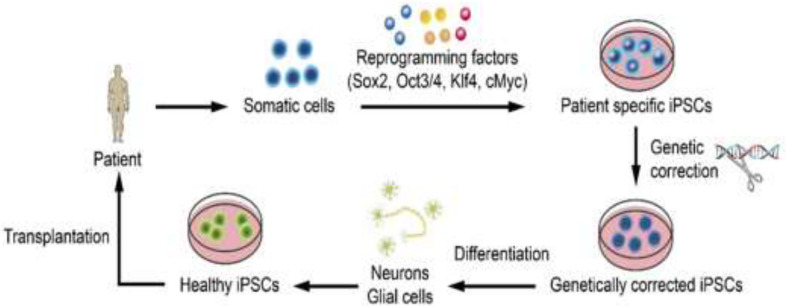
Steps of iPSC-based therapy. First, somatic cells are collected from the patient. Next, somatic cells are reprogrammed into iPSCs by the introduction of reprogramming factors. Thereafter, genetically corrected iPSCs are generated by genome editing. The corrected iPSCs differentiate into neurons or glial cells. Healthy iPSCs are obtained via quality assessments. Finally, cell therapy can be achieved by transplanting the cells into a patient with ischemic stroke (Alessandrini et al., 2019; Farkhondeh et al., 2019; Yasuhara et al., 2020).

iPSCs are derived directly from the connective tissue of patients through a small biopsy and exhibit the same properties as ESCs, thereby overcoming the problems related to immune rejection and bypassing the need for embryos, to avoid ethical issues. Therefore, iPSCs can be generated in a patient-matched manner, each individual having his/her own PSC line. First, somatic cells, such as fibroblasts, are collected from the patient. Next, somatic cells are reprogrammed into iPSCs by the introduction of reprogramming factors. Thereafter, genetically corrected iPSCs are generated by genome editing. The corrected iPSCs differentiate into neurons or glial cells. Healthy iPSCs are obtained via quality assessments. Finally, cell therapy can be achieved by transplanting the cells into a patient with ischemic stroke (Alessandrini et al., 2019; Farkhondeh et al., 2019; Yasuhara et al., 2020; Figure 2). However, there are several obstacles and limitations related to iPSC-based therapy that need to be overcome before clinical application.

#### Teratoma Formation

Although iPSC-NPCs could survive and migrate toward the lesion area, and were shown to alleviate the dysfunction induced by ischemic stroke, without tumor formation, during a 4-month period after transplantation (Jirak et al., 2019), the risk of tumorigenesis from iPSCs remains a major constraint for clinical application. A previous study had shown that direct injection of iPSCs into the ischemic brain caused teratoma formation in 100% of MCAO model rats 4 weeks after transplantation (Chen et al., 2010). In addition, the transplanted iPSCs expanded in the brain of post-ischemic mice and formed tumors that were larger than those formed in the sham-operated group (Kawai et al., 2010). The initial induction process of iPSCs required the use of a retrovirus carrying reprogramming factors, including Sox2, Oct3/4, Klf4, and cMyc, the latter two being oncogenes that may cause tumorigenicity in the host (Sun et al., 2010). Therefore, it is essential to strictly evaluate whether iPSCs and their derivatives could induce tumor growth in the host and to protect the same from happening.

Other studies have shown that, out of Oct4 and Klf4 as small-molecule compounds, Oct4 alone is sufficient to induce iPSCs (Shi et al., 2008; Kim et al., 2009). Some non-viral methods, such as plasmid vectors, minicircular DNA carriers, and proteins have been developed as a safer approach to generate iPSCs successfully (Cho et al., 2010; Okita et al., 2010; Rhee et al., 2011; Okano et al., 2013). Purification of cells before transplantation and some small-molecule inhibitors can effectively reduce the potential tumorigenicity of iPSCs. Quercetin/YM155-induced selective cell death (Lee et al., 2013) and pluripotent cell-specific inhibitors (PluriSIns) (Ben-David et al., 2013) were shown to be sufficient to completely inhibit teratoma formation by efficiently eliminating residual undifferentiated cells, which could increase the safety of iPSC-based treatments. The predifferentiation of iPSCs and establishment of long-term self-renewing neural cell lines are other effective strategies proposed to reduce the risk of tumor formation (Oki et al., 2012).

#### Low Induction Efficiency

Several solutions have also been put forward to overcome the problem of low iPSC induction rate. For example, stress-mediated p38 activation may enhance the low induction rate of iPSCs, and hyperosmosis has been reported to promote not only cell reprogramming but also iPSC generation (Xu et al., 2013). Expression of the mouse miR302/367 cluster could rapidly and successfully reprogram iPSCs, and the induction rate of this microRNA-based reprogramming method was shown to be 100 times greater than that of the original method (Anokye-Danso et al., 2011). As mentioned above, Sox2, Oct3/4, Klf4, and cMyc are of great importance in the generation of iPSCs. However, silencing of P53 with small interfering RNA and addition of undifferentiated embryonic cell transcription factor 1 (UTF1) could increase the production efficiency of iPSCs by 100 times, even if the oncogene c-Myc was removed from the combination (Nakagawa et al., 2008; Zhao et al., 2008). Another study confirmed that some small-molecule compounds can also effectively improve the induction efficiency. For instance, both DNA methyltransferase and histone deacetylase (HDAC) inhibitors play an important role in reprogramming efficiency. Valproic acid, an HDAC inhibitor, increased the reprogramming efficiency of iPSCs by more than 100 times, which could also be achieved without introduction of the oncogene c-Myc (Huangfu et al., 2008). Collectively, these findings highlighted the importance of achieving safety of the host after transplantation while ensuring a high induction rate.

#### Immune Rejection

Immune-like cells, with large nuclei, cluster around the transplanted cells, indicating immune rejection to continue after 6 weeks of transplantation (Gomi et al., 2012). Abnormal gene expression in some cells differentiated from iPSCs exhibited the potential to induce a T cell-dependent immune response in syngeneic recipients. This immunogenicity may be caused by the abnormal expression of antigens between ESCs and iPSCs due to epigenetic differences, leading to the destruction of peripheral immune tolerance (Zhao et al., 2011). The emergence of zinc finger nucleases, transcription activator–like effector nucleases (TALENs), and the clustered regularly interspaced short palindromic repeat (CRISPR)-associated protein 9 (Cas9) system, as powerful gene-editing tools, has now made it possible to modify genes efficiently and accurately. Genomic correction of mutant iPSCs may lead to healthy differentiated cells, thus avoiding the risk of immune rejection (Kim and Kim, 2014; Hockemeyer and Jaenisch, 2016; Ben Jehuda et al., 2018). In future, more attention should be paid to the occurrence of an immune response during iPSC transplantation, and additional research should be conducted to assess and avoid immune rejection.

Apart from the above limitations, overexpression of stromal cell-derived factor 1 alpha (SDF-1α) or optochemogenetic stimulation of iPSC-NPCs may be an effective strategy to enhance endogenous neurovascular regeneration and functional recovery after ischemic stroke (Chau et al., 2017; Yu et al., 2019). A study conducted in 2010 had found that subdural iPSCs-FG can significantly reduce the total infarct volume and improve the behavior of rats with MCAO, indicating subdural transplantation of iPSCs-FG to be a safer administration route (Chen et al., 2010). Transplantation of MSCs through the intra-arterial route increased the risk of cerebral lesions and did not improve functional recovery in a transient cerebral ischemia rat model (Argibay et al., 2017), whereas the intra-arterial transfer of MSCs/neurogenin-1 could block neuronal cell death and inflammation to ultimately improve functional recovery (Kim et al., 2020).

## Conclusion and Future Prospects

The explosion of research on stem cell therapy and its characteristics has ushered in a new era for the treatment of ischemic stroke. At the same time, iPSC technology has also become increasingly mature for the treatment of ischemic stroke, achieving considerable results in preclinical models, thereby bypassing the ethical and immune rejection concerns related to the use of ESCs. At present, iPSCs are considered a promising tool for clinical treatment, and are expected to be practically applicable in the near future. To realize this therapy, we should strive to overcome the remaining obstacles, and conduct wider and more in-depth clinical trials. Importantly, a consensus is required for optimizing the methods for cultivation and differentiation of iPSCs. In addition, different iPSC lines may vary in their ability to differentiate into different cell lines, a process that warrants further attention. The development and evaluation of various strategies may offer a promising solution to overcome the tumorigenicity and low efficiency of iPSC induction. Appropriate transplantation methods can improve the survival rate of transplanted cells and further strengthen the therapeutic effect. When choosing transplantation methods, in addition to determining the transplantation time and route, it would be necessary to determine the differentiation state, transplantation administration, dose, and auxiliary status before transplantation.

In summary, with rapid progress and improvement in the technology of iPSCs, and gaining better understanding of the reprogramming and therapeutic mechanisms, the ultimate goal of providing iPSCs safely and with relatively high efficiency to patients with ischemic stroke may become a reality in near future.

## Author Contributions

RD, YG, and RH searched for relevant literature and drafted the manuscript together. LJ and YL searched for relevant literature. YY and ZG revised the manuscript critically. TL and CZ were involved in preparing the figures and tables. LL and YJ provided professional guidance for this review and performed a final check of the manuscript. All authors contributed to the review of this manuscript and approved the submitted version.

## Conflict of Interest

The authors declare that the research was conducted in the absence of any commercial or financial relationships that could be construed as a potential conflict of interest.
